# Quantifying the impact of between-study heterogeneity in multivariate meta-analyses

**DOI:** 10.1002/sim.5453

**Published:** 2012-07-04

**Authors:** Dan Jackson, Ian R White, Richard D Riley

**Affiliations:** a MRC Biostatistics UnitCambridge, U.K.; b School of Health and Population Sciences, University of BirminghamBirminghan, U.K.

**Keywords:** generalised variance, meta-regression, multivariate methods, random effects models

## Abstract

Measures that quantify the impact of heterogeneity in univariate meta-analysis, including the very popular *I*^2^ statistic, are now well established. Multivariate meta-analysis, where studies provide multiple outcomes that are pooled in a single analysis, is also becoming more commonly used. The question of how to quantify heterogeneity in the multivariate setting is therefore raised. It is the univariate *R*^2^ statistic, the ratio of the variance of the estimated treatment effect under the random and fixed effects models, that generalises most naturally, so this statistic provides our basis. This statistic is then used to derive a multivariate analogue of *I*^2^, which we call 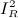. We also provide a multivariate *H*^2^ statistic, the ratio of a generalisation of Cochran's heterogeneity statistic and its associated degrees of freedom, with an accompanying generalisation of the usual *I*^2^ statistic, 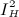. Our proposed heterogeneity statistics can be used alongside all the usual estimates and inferential procedures used in multivariate meta-analysis. We apply our methods to some real datasets and show how our statistics are equally appropriate in the context of multivariate meta-regression, where study level covariate effects are included in the model. Our heterogeneity statistics may be used when applying any procedure for fitting the multivariate random effects model. Copyright © 2012 John Wiley & Sons, Ltd.

## 1. Introduction

Meta-analysis, the statistical process of pooling the results from separate studies concerned with the same treatment or issue, is a well-established tool in medical statistics. Meta-analysis does, however, present both computational and conceptual difficulties associated with between-study heterogeneity. This additional source of variation is usually modelled using the random effects model [1–3]. Here, the between-study variance allows for any apparent over-dispersion of studies' results.

If the between-study variance is assumed to be zero, then the model is conventionally referred to as the fixed effects model. This model simplifies the resulting interpretations, and eases computation, but the assumption of no between-study variation seems generally implausible, unless it is known that the studies are performed in the same way and involve individuals sampled from the same population.

Tests for the presence of heterogeneity exist but have low power [4], and their use to choose between fixed and random effects models is generally discouraged [4]–[6]. Statistics that quantify the impact of heterogeneity have been proposed as an alternative to this testing [5], and *I*^2^, which describes the proportion of variability in point estimates that is due to heterogeneity rather than within-study sampling error, is now almost always provided in addition to the results from the standard heterogeneity test. In addition to *I*^2^, Higgins and Thompson [5] suggested two further heterogeneity statistics, *H*^2^ and *R*^2^; we describe all three of these heterogeneity statistics in Section 4. The very popular *I*^2^ has, however, recently received criticism from Rücker *et al*. [7] who show how this quantity depends on the size of the studies. Although this dependence is clearly explained by Higgins and Thompson [5], this has resulted in some questioning its use. Our position is that the heterogeneity statistics are useful descriptive statistics when used in conjunction with the estimate of the between-study variance and all the other usual inferential statistics, such as the pooled effect.

More recently, multivariate meta-analysis [8–11] has become established. Here, multiple study outcomes are combined in a single multivariate analysis, to account for their correlation. For example, diagnostic test studies provide estimates of sensitivity and specificity, which are usually negatively correlated between studies. The multivariate methods are generalisations of their more commonly used univariate counterparts and possess many advantages, but they also have their limitations [10]. The most commonly referred to advantage of the multivariate approach is the ‘borrowing of strength’ that can occur as a result of the utilisation of correlation. This applies to both the pooled estimates and the between-study variance estimates. For example, it has been shown that the multivariate model gives a smaller mean-square error and, on average, standard error of the pooled estimates than the univariate method [12]. Now that multivariate meta-analysis has arrived, and the importance of univariate measures for quantifying the impact of heterogeneity is well understood, an obvious missing component is the development of appropriate multivariate measures of heterogeneity. Although there has been methodological development in the form of White's *I*^2^ statistics [13], as described in Section 5.4, the intention here is to develop some multivariate heterogeneity statistics that are either generalisations or analogues of the established univariate statistics.

We follow Higgins and Thompson [5] by conceptualising the heterogeneity statistics as quantifying the *impact* of between-study heterogeneity. By this, we refer to the impact of both the between-study variances and correlations, that is, the entire between-study covariance matrix. Testing the null hypothesis that there is no between-study variation, and estimating the magnitude of this, are related procedures that address different statistical questions. We focus on the impact that the heterogeneity has on the precision of the estimated treatment effect, by comparing the precision of estimates from a random effects meta-analysis to those from a fixed effects analysis. For example, if the random effects model provides pooled estimates with similar precision to those from a fixed effects meta-analysis, then the heterogeneity is considered to have little impact.

Although we focus on the relative precision of estimates, the random effects model gives smaller studies greater weight so that the heterogeneity can also impact directly on the point estimate of treatment effect if small studies provide estimates that differ to those from larger ones. If this is the case, then a special investigation is required because the various heterogeneity measures do not attempt to quantify the impact of small study effects or publication bias. This type of issue is exacerbated in the multivariate setting because, in addition to these possibilities, the borrowing of strength may also depend on the amount of heterogeneity. We therefore might anticipate that the multivariate methods provide greater capacity for random and fixed effects analyses to provide notably different point estimates. Here, we do not attempt to quantify the impact of heterogeneity on the location of the point estimate of treatment effect, or the amount of borrowing of strength afforded by multivariate rather than univariate analyses, but these are also important issues and may form the subject of future work.

The unfashionable (because *I*^2^ has become so popular) *R*^2^ statistic, the ratio of the variances of the pooled treatment effect under the random and fixed effects models, is the most natural to extend multivariately. We begin with this as our basis and define an *R* statistic; univariately, *R* is the square root of the established *R*^2^ statistic. We then apply this to define a multivariate *I*^2^ statistic, 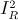. Our 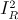 statistic describes the proportion of the variation of the pooled vector of estimates under the random effects model, which is due to between-study variation. We also provide a multivariate *H*^2^ statistic, the ratio of a generalisation of Cochran's heterogeneity statistic and its associated degrees of freedom, and an accompanying generalisation of the usual *I*^2^ statistic 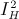. The *R* statistic is based on the covariance matrix of the estimated treatment effects, and *H*^2^ is based on the residuals from a fixed effects model fit. Hence, they can also be used in the context of multivariate meta-regression [14], where covariate effects are included, and for any procedure for fitting the random effects model.

We set out the rest of the paper as follows. We briefly present the univariate and multivariate models in Section 2 and apply these to our sample datasets in Section 3. We present the univariate heterogeneity statistics in Section 4. In Section 5, we derive our multivariate measures; and in Section 6, we apply our proposed measures, and their univariate counterparts, to our examples. We explain how our measures of heterogeneity may be used in the context of multivariate meta-regression in Section 7 and conclude with a discussion in Section 8.

## 2. Univariate and multivariate random effects meta-analysis

We present the multivariate random effects meta-analysis model and explain how this reduces to the usual univariate model in a single dimension. We denote the vector of outcomes (or estimates) for the *i*th study as **Y**_*i*_. For example, **Y**_*i*_ may be a vector containing the log hazard ratios of overall and disease-free survival.

The entries of **Y**_*i*_ may be correlated, and it is assumed that


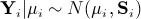


where *N* denotes a multivariate normal distribution, *μ*_*i*_ is the true underlying effect for the *i*th study and **S**_*i*_ is the covariance matrix of **Y**_*i*_. The matrices **S**_*i*_ are referred to as the within-study covariance matrices; their entries are estimated in each study in practice but regarded fixed and known when pooling the studies' results. Estimating the within-study covariances or correlations, to provide the off diagonal entries of the **S**_*i*_, is often difficult in practice, but a variety of approaches are possible [10].

The multivariate random effects model allows for the possibility that the *μ*_*i*_ may vary from one study to the next and further assumes that


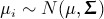


where *μ* is the (overall) treatment effect vector and ***Σ*** is the between-study covariance matrix. Marginally, this provides the conventional multivariate random effects meta-analysis model


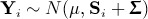
(1)

where the **Y**_*i*_ are further assumed to be independent. If all entries of ***Σ*** are constrained to zero, then the model reduces to a fixed effects model.

The conventional univariate random effects model is simply the marginal distribution of the first (say) study outcome. In one dimension, and written in the more usual univariate notation, this means that each study provides a univariate 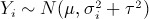. If all within-study and between-study correlations are assumed to be zero, then the multivariate random effects model is the collection of the univariate random effects models for each of the study outcomes.

The standard procedure for making inferences about the treatment effect approximates the true between-study covariance with 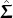 [10]. After performing this estimation, the pooled (maximum likelihood) estimates are given by


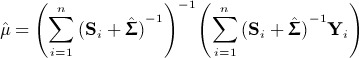
(2)

where *n* is the number of studies, and these estimates are approximately normally distributed with covariance matrix


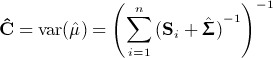
(3)

Alternatively, the covariance matrix can be obtained from the observed Fisher information matrix, and Stata's *mvmeta* [13] uses this method as its default. Equations (2) and (3) require an estimated between-study covariance matrix, and a variety of estimates are available [10]. A fixed effects model is fitted by constraining all entries of 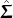 to zero in (2) and (3). If some studies have missing outcomes, then, assuming that these are missing at random, such studies can be incorporated into these matrix solutions by allocating notional estimates with very large within-study variances and corresponding within-study correlations of zero, or better by modifying these equations to use the marginal model from (1) for the observed data. If inferences for particular subsets of outcomes are required, then these are obtained from the corresponding marginal distributions from (2) and (3). In one dimension, this reduces to the usual univariate formulae, that is,(2) and (3) reduce to 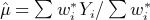 and 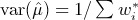 where, in the more usual univariate notation, 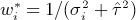.

## 3. Examples

In this section, we apply the methods described in Section 2 to some contrasting examples and informally assess the impact of the between-study heterogeneity on the precision of the pooled estimates. We used the Stata program *mvmeta* with its defaults throughout. Hence, we adopted the restricted maximum likelihood estimation of the between-study covariance matrix and used the entire observed Fisher information matrix (including the variance components) to compute the covariance matrix of the treatment effect parameters. White [13] described in detail the alternative estimation methods, but we used the defaults here because these acknowledge the uncertainty in the estimation of the between-study covariance matrix and because restricted maximum likelihood is well established in multivariate meta-analysis. Hence, the uncertainty in the between-study covariance matrix is reflected in the results that follow, but we do not wish to imply that this is fully taken into account.

### 3.1. Example 1: the periodontal data

The periodontal data from Berkey *et al*. [15, 16] involve five studies providing the mean difference between surgical and non-surgical procedures for treating periodontal disease, with improvement in probing depth and improvement in attachment level as the two endpoints of interest (measured in mm one year after treatment). We show the data in Table I, and the within-study covariances are known. The within-study correlations are positive as one might expect because both outcomes are associated with positive patient outcomes.

**Table I:** Periodontal data, providing the mean difference between surgical and non-surgical procedures for treating periodontal disease, with improvement in probing depth and improvement in attachment level as the two end points of interest (measured in mm, one year after treatment)

Study	*Y*_1_	*S*_11_	*Y*_2_	*S*_22_	*S*_12_
1	0.47	0.0075	− 0.32	0.0077	0.0030
2	0.20	0.0057	− 0.60	0.0008	0.0009
3	0.40	0.0021	− 0.12	0.0014	0.0007
4	0.26	0.0029	− 0.31	0.0015	0.0009
5	0.56	0.0148	− 0.39	0.0304	0.0072

We show the results from the univariate and multivariate random effects meta-analyses in Table II. The univariate and multivariate analyses are in good agreement and indicate that the surgical procedure improves probing depth by about 0.35 mm more than the non-surgical procedure, but that the non-surgical procedure improves attachment level by a similar amount than the surgical procedure. There is, however, a large amount of between-study variation whose impact is clear from the covariance matrix of the estimated treatment effects from random and fixed effects multivariate meta-analyses. These covariance matrices are, using *mvmeta*'s defaults,


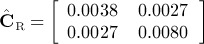


and


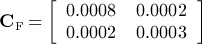


respectively. Because the within-study covariance matrices are regarded as known, **C**_F_ is treated as a constant, and is given by (3) with all entries of 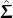 set to zero. From an inspection of the relative magnitudes of the diagonal entries of these two covariance matrices, it appears that heterogeneity has a greater impact on the second outcome than on the first. Because the univariate and multivariate analyses are in such good agreement, it might be anticipated that the conventional univariate heterogeneity statistics (Section 4) will give a good indication of the impact of heterogeneity on the marginal inferences for both outcomes. The best way to quantify the impact of heterogeneity on the joint inferences of both outcomes, when using the multivariate model, is less clear however.

**Table II:** Parameter estimates for the periodontal data in example 1 using the random effects model

	*μ*_1_	*μ*_2_	Σ_11_	Σ_22_	Σ_12_
Univariate	0.361 (0.060)	− 0.346 (0.089)	0.012	0.033	—
Multivariate	0.353 (0.061)	− 0.339 (0.089)	0.012	0.033	0.012

We show the standard errors of the treatment effect parameters in parentheses.

### 3.2. Example 2: the sleep data

Our second example is the sleep data of McDaid *et al.* [17]. This is another bivariate example, and the full dataset is available from the authors on request. Here, we have 26 studies providing the mean effect of treatment for obstructive sleep apnoea/hypopnoea syndrome in terms of change in Epworth Sleepiness Scale (*Y*_1_) and change in systolic blood pressure (*Y*_2_). Twenty three studies give information on *Y*_1_, but only 10 studies give information on *Y*_2_, so there is a considerable scope for borrowing strength for *μ*_2_ in a multivariate meta-analysis. The within-study correlations, and hence the off diagonal entries of the within-study covariance matrices, are unknown but *Y*_1_ and *Y*_2_ may be positively correlated because both a lack of sleep and high blood pressure may be associated with elevated stress levels. Here, we perform an illustrative multivariate analysis assuming all within-study correlations are 0.4, which provide modest correlations within studies; other values could also be explored in a sensitivity analysis, and other options for dealing with the unknown within-study correlations are possible [10]. We show the results from the univariate and multivariate random effects meta-analyses in Table III. Here, the estimated between-study correlation is one so that the estimated random effects lie at the edge of their parameter space, which has consequences for the estimation [12].

**Table III:** Parameter estimates for the sleep data in example 2 using the random effects model

	*μ*_1_	*μ*_2_	Σ_11_	Σ_22_	Σ_12_
Univariate	− 2.68 (0.41)	− 3.03 (1.29)	2.56	5.91	—
Multivariate	− 2.49 (0.39)	− 4.64 (1.34)	2.52	13.70	5.87

We show the standard errors of the treatment effect parameters in parentheses.

The results in Table III suggest that the treatment for obstructive sleep apnoea/hypopnoea syndrome is effective for both outcomes. Again, there is considerable heterogeneity whose impact is shown by the covariance matrix of the estimated treatment effects from random and fixed effects multivariate meta-analyses


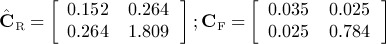


The univariate and multivariate estimates are very different, particularly for the second outcome (Table III). In particular, the estimate of the marginal between-study variance of the second outcome is sensitive to the choice between univariate or multivariate meta-analyses. Thus, the conventional univariate heterogeneity statistics cannot be expected to give a good indication of the impact of heterogeneity on the marginal inferences for the second outcome when using the multivariate model, or the joint inferences for both outcomes.

### 3.3. Example 3: the prognostic value of MYCN and chromosome 1p

This is similar to the example used by Riley [11], but here, we include 73 observational studies that examine two effects: overall and disease-free survival. The full dataset is available from the authors upon request. These studies assess the prognostic value of up to two factors, MYCN and chromosome 1p, in patients with neuroblastoma and were also used as an example by Jackson *et al.* [10]. Patients either have a ‘high’ or ‘low’ level of MYCN and either chromosome 1p presence or deletion. Studies provide up to four estimates of effect, each of which is an estimated unadjusted log hazard ratio of survival, either of the high relative to the low level group of MYCN, or chromosome 1p deletion to its presence. The within-study correlations are unknown to the authors but are taken here in an illustrative analysis as 0.7. The log hazard ratios are highly likely to be positively correlated within and between studies across all four outcomes, because MYCN and chromosome 1p are often highly correlated in a patient, whereas overall and disease-free survival are inherently correlated by their definitions. We used the variables *Y*_1_ and *Y*_2_ to denote the log hazard ratio for disease-free survival for high to low MYCN and the deletion to the presence of chromosome 1p markers, respectively; *Y*_3_ and *Y*_4_ denote the corresponding overall survival log hazard ratios. Thirty four, 8, 50 and 10 studies report *Y*_1_ to *Y*_4_, respectively.

The average log hazard ratio estimates in Table IV are significantly greater than zero; and hence, chromosome 1p and MYCN have a prognostic value for both disease-free and overall survival. The heterogeneity also has notable impact for this example and


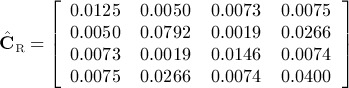


and


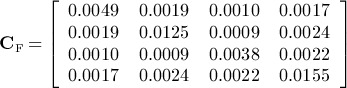


**Table IV:** Parameter estimates for the MYCN and chromosome 1p data in example 3 using the random effects model

	*μ*_1_	*μ*_2_	*μ*_3_	*μ*_4_		
Univariate	1.58 (0.14)	1.33 (0.29)	1.69 (0.13)	1.26 (0.23)		
Multivariate	1.59 (0.11)	1.18 (0.28)	1.71 (0.12)	1.15 (0.20)		

	Σ_11_	Σ_22_	Σ_33_	Σ_44_		
Univariate	0.33	0.44	0.37	0.22		
Multivariate	0.31	0.57	0.45	0.39		

	Σ_12_	Σ_13_	Σ_14_	Σ_23_	Σ_24_	Σ_34_
Univariate	—	—	—	—	—	—
Multivariate	0.11	0.36	0.28	0.01	0.36	0.27

The parameters *μ*_1_ and *μ*_2_ denote the average log hazard ratios for disease-free survival for high to low MYCN and the deletion to the presence of chromosome 1p, respectively. Parameters *μ*_3_ and *μ*_4_ denote these same hazard ratios for overall survival. We show the standard errors of the treatment effect parameters in parentheses.

The estimates of the marginal between-study variances are quite sensitive to the choice between univariate and multivariate meta-analyses. For an example such as this, in a relatively high dimension and where there is much missing data and borrowing of strength occurs [10], there is little to provide reassurance that the conventional univariate heterogeneity statistics will adequately quantify the impact of heterogeneity in the multivariate analysis. Furthermore, particular subsets of the treatment effects are jointly of interest, such as those relating to the two types of survival and the two markers separately. Methods for quantifying the impact of heterogeneity for more than a single outcome are particularly valuable here, and something more sophisticated than the established univariate statistics is required.

## 4. Conventional univariate measures of heterogeneity

Higgins and Thompson [5] originally defined three univariate measures of the impact of heterogeneity, *R*^2^, *H*^2^ and *I*^2^, which we will extend multivariately. Variations have, however, subsequently been suggested [18]. We use the univariate notation in this section, 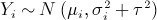. We use 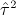 to denote DerSimonian and Laird's [1] estimate of the between-study variance and *Q* to denote Cochran's heterogeneity statistic [1, 18]. Hence, we have


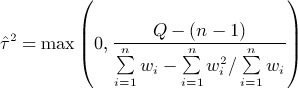


where


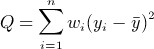



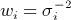
 and 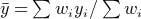. Higgins and Thompson define


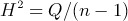



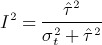


where 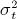 is the ‘typical within-study variance’


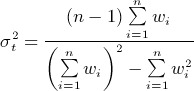


and *I*^2^ = (*H*^2^ − 1) / *H*^2^, where *I*^2^ is truncated to zero if (*H*^2^ − 1) / *H*^2^ < 0. Higgins and Thompson suggest that ‘mild heterogeneity’ might correspond to *I*^2^ < 0.3 and ‘notable heterogeneity’ might correspond to substantially more than *I*^2^ = 0.5, but these are only tentative suggestions. Overlapping intervals for *I*^2^ have subsequently been provided to avoid over-interpretation of *I*^2^ statistics [6]. Higgins and Thompson's definition of *R*^2^ is equivalent to defining


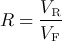


where *V*_R_ and *V*_F_ are the length of the confidence intervals for the treatment effect that arise from the random and fixed effects model, respectively, assuming that standard normal quantiles are used to construct both intervals; *t* distribution quantiles are sometimes suggested when using random effects models in meta-analysis [14]. Higgins and Thompson define *R* in terms of the treatment effect's standard errors under the random and fixed effects models, but it is the relative length of the confidence intervals that generalises multivariately. We use the notation *V*_R_ and *V*_F_ because multivariately, these quantities become generalised notions of the volumes of confidence regions that arise from the two models. This involves a slight clash of notation with Higgins and Thompson who use *v*_R_ and *v*_F_ to denote the variance of the estimates of treatment effect. Simulation-based [19] and analytical [18] investigations of the univariate measures of heterogeneity have been performed.

## 5. Multivariate measures of heterogeneity

From a comparison of the 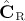 and **C**_F_ obtained from multivariate meta-analyses for our three examples, and our interpretation of the impact of heterogeneity as referring to the relative precision of estimates resulting from random and fixed effects multivariate meta-analyses, it is clear that the heterogeneity has a considerable impact for all three examples. The aim is to quantify this impact.

### 5.1. Multivariate *R* statistic

The univariate *R* statistic is perhaps the univariate heterogeneity statistic that is most naturally extended to achieve this aim. Let *p* denote the number of treatment effect parameters that the heterogeneity statistic applies to, which for now we assume is the dimension of the meta-analysis, and we also assume that standard normal quantiles are used to construct both random and fixed effects confidence regions. As alluded to earlier, we denote the volumes of the confidence regions for all *p* outcomes in *μ* that arise from the random and fixed effects models as *V*_R_ and *V*_F_, respectively, and we define


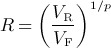
(4)

This *R* statistic reduces to the usual univariate meta-analysis measure when *p* = 1. In one dimension, *V*_R_ and *V*_F_ are the lengths of the random and fixed effects confidence intervals; and in two and three dimensions, they are areas and volumes, respectively. In four or more dimensions, *V*_R_ and *V*_F_ are generalised notions of volumes of the random and fixed effects confidence regions. We calculate *R* as shown in equation (4) irrespective of the method used to estimate the between-study covariance matrix, the form that this takes, or the method used to obtain 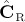 once the between-study covariance has been estimated.

One interpretation of *R* is as the ratio of the geometric means of the standard errors of the random and fixed effects estimates of treatment effect that result when their normal approximations are reparameterised in terms of the rotated co-ordinate system where the principal axes are used. In linear algebra, this is referred to as writing the normal approximation's associated quadratic form in its *standard position*. *R* is therefore an average ratio of the lengths of random and fixed effects confidence intervals across all outcomes.

We show in Appendix A that (4) can be conveniently written as


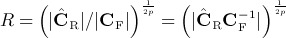
(5)

where | **C**_*a*_ | is the determinant of **C**_*a*_. The matrices 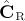 and **C**_F_ are obtained when fitting the random and fixed effects models, respectively, so that the measure (5) is easy to obtain from the standard output from statistical software. Equation (5) provides further interpretation of our *R* statistic because the determinant of a covariance matrix is referred to as the generalised variance. This is considered to be a good scalar dispersion statistic for multivariate data. Equation (5) shows that *R* is a function of the ratio of the generalised variances of the estimated treatment effect under the random and fixed effects models.

### 5.2. Multivariate 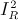 statistic

It is the univariate *I*^2^ statistic that is most commonly used in practice, so a multivariate version of this can be expected to be especially valuable. Higgins and Thompson [5] provide the univariate relationship *I*^2^ = (*H*^2^ − 1) / *H*^2^ (their Equation (10)) and show that *H*^2^ and *R*^2^ measure similar quantities. This suggests the definition


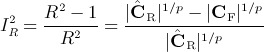
(6)

The quantity 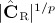 is the square of the geometric mean of the standard errors of the estimated treatment effect from the random effects model when its associated quadratic form is in its standard position. Hence, 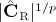 is an average variance resulting from the random effects model, and 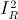 is the proportion of this variance, which is explained by between-study heterogeneity. The proposed multivariate 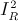 is therefore an analogue of the univariate *I*^2^ statistic, with a similar but not identical interpretation to its established univariate counterpart. If all studies are the same ‘size’ (**S**_*i*_ = **S**_1_ for all *i*), then (6) simplifies to the usual *I*^2^ statistic univariately, but this is not the case in general. We return to the apparent issue of truncating potentially negative 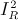 statistics to zero so that these cannot be negative, just as in the univariate case when equating *I*^2^ = (*H*^2^ − 1) / *H*^2^ as described in Section 4, in Section 5.5.

### 5.3. Multivariate *H*^2^ and 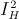 statistics

A multivariate *H*^2^ statistic is also desirable, primarily to provide a direct extension of the univariate *I*^2^ statistic. The univariate *H*^2^ statistic is defined directly in terms of *Q* as shown in Section 4. The difficulty in using the matrix *Q* proposed by Jackson *et al.* [14] for the purposes of quantifying heterogeneity multivariately is discussed when presenting White's *I*^2^ statistics in Section 5.4 but an alternative multivariate generalisation of *Q* is


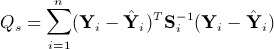
(7)

where 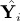 is the fitted value from the fixed effects model. The subscript *s* emphasises that *Q*_*s*_ is a scalar, a *χ*^2^ test statistic that can be used to test the null hypothesis that there is no between-study heterogeneity. We make the obvious multivariate generalisation


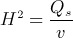


where *v* denotes the degrees of freedom of *Q*_*s*_, that is, the total number of univariate estimates minus the dimension of the meta-analysis. We can then define an *I*^2^ statistic on the basis of *H*^2^


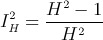


as another possible measure of heterogeneity. The *H*^2^ statistic retains its interpretation from the univariate case and the multivariate *H*^2^ and 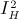 statistics simplify to the conventional *H*^2^ and *I*^2^ statistics univariately. We suggest that 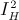 is truncated to zero if (*H*^2^ − 1) / *H*^2^ < 0, following the usual convention in the univariate case.

The quantity that the multivariate *H*^2^ statistic estimates is derived in Appendix B.

### 5.4. White's *I*^2^ statistics

Another way to generalise the univariate *I*^2^ statistic multivariately is to exploit the fact that the univariate *I*^2^ may be written in terms of *Q*, as max(0,(*Q* − (*n* − 1)) / *Q*) [18]. A multivariate *Q* matrix has recently been suggested, which can be used to estimate the between-study covariance matrix. Jackson *et al.* [14], however, ‘note that the proposed *Q* matrix is not based on matrix operations’. Hence, it is not clear that any standard matrix operation would be capable of transforming this *Q* matrix into a heterogeneity statistic. It is, at best, extremely difficult to base any truly multivariate measure of heterogeneity on this *Q* matrix; and hence, *I*^2^ is hard to generalise multivariately in this way.

Despite these issues, White [13] has recently proposed multivariate *I*^2^ statistics that reduce to the univariate measure in one dimension. This defines *I*^2^ statistics as the ratios of the estimated between-study variances and the sum of these variances and ‘typical within-study variances’, where these within-study variances are the ratio of coefficients from the recently proposed multivariate DerSimonian and Laird estimating equations [14]. This may not be inappropriate, but White's *I*^2^, just like the conventional univariate one, depends crucially on this typical variance. Any method for computing such a variance becomes increasingly problematic as the dimension of the meta-analysis increases. Furthermore, White's *I*^2^ statistics do not truly reflect the multivariate nature of the model fit, or the association between the estimates, because they are merely functions of the estimated marginal between-study variances and the within-study covariance matrices. Despite these limitations, we will compare White's *I*^2^ statistics with those that we develop here.

### 5.5. Multivariate *R* and 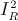 statistics for subsets of the outcomes

One might be especially interested in quantifying heterogeneity for a subset of the outcomes; for example, some effects might be considered to relate to primary trial outcomes. Both the *R* and 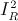 measures can be easily applied to just a subset of the estimated effects by taking the corresponding submatrices of 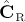 and **C**_F_ and by performing the calculations shown in (5) and (6) where *p* is taken as the dimension of the subset of the outcomes under consideration. We show in Appendix C that our multivariate *R* statistics, for all or just some of the outcomes, are greater than or equal to one if (3) is used to obtain the variance of the pooled estimates; and hence, the corresponding 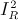 statistics are greater or equal to zero. Therefore, no truncation of 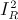 statistics to zero in such instances is ever required. Because other methods for obtaining the variance of the pooled estimates, for example, using the observed Fisher information matrix, approximate the variance (3), we anticipate that the truncation out of 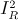 statistics is not likely to be a common occurrence irrespective of the procedure used, but we suggest that this is performed where necessary.

It is also possible that one might be interested in quantifying heterogeneity for certain contrasts or linear combinations of the effects. For example, a linear combination of sensitivity and specificity might be important in determining the value of a diagnostic test [20]. Upon obtaining the covariance matrix of the fixed and random effects models' estimates of these linear combinations, these could also be used in (5) and (6) to obtain multivariate *R* and 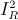 statistics for any linear combinations of interest.

However, the properties of *Q*_*s*_, if we instead sum over a subset of the outcomes (and so use the corresponding subvectors of **Y**_*i*_ and 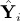, and submatrices of 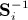, when computing (7)) are not clear. We therefore propose that *H*^2^ and 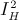 be used to quantify the heterogeneity for all treatment effect parameters, whereas *R* and 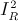 can be used for all, or a subset of, these parameters as desired. We provide a summary of all the various heterogeneity measures and in Table V, where the interpretations of the conventional univariate heterogeneity statistics are as described by Higgins and Thompson [5].

**Table V:** A Summary of the existing and proposed heterogeneity statistics

Statistic	Interpretation	Available for each outcome separately?	Available for all outcomes jointly?
Univariate *I*^2^	The proportion of total variation in the estimates of treatment effect that is due to heterogeneity between studies in a univariate meta-analysis	Yes	No
Univariate *R*	The inflation in the confidence interval for a single summary estimate under a random effects model compared with a fixed effects model in a univariate meta-analysis	Yes	No
Univariate *H*^2^	The relative excess in *Q* over its degrees of freedom in a univariate meta-analysis	Yes	No
White's *I*^2^	Proportions of total marginal variation in the estimates of treatment effect that are due to heterogeneity between studies in a multivariate meta-analysis	Yes	No
Multivariate *R*	The inflations in the confidence regions for pooled estimates under a random effects model compared with a fixed effects model in a multivariate meta-analysis	Yes	Yes
Multivariate 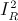	The proportion of variation in the pooled estimates of treatment effect that is due to heterogeneity between studies in a multivariate meta-analysis	Yes	Yes
Multivariate *H*^2^	The relative excess in *Q*_*s*_ over its degrees of freedom in a multivariate meta-analysis	No	Yes
Multivariate 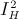	A direct generalisation of the univariate *I*^2^ statistic in a multivariate meta-analysis	No	Yes

## 6. Applying the proposed heterogeneity statistics to our examples

We summarized the various heterogeneity measures for our three example datasets in Tables VI, VII and VIII, where we continue to use *p* to denote the number of treatment effect parameters that the heterogeneity statistics apply to. We present all the heterogeneity statistics in our tables but restrict our interpretations to the *I*^2^ statistics. This is because the univariate *I*^2^ statistic is easily the most popular, but the reader may also use the *R* and *H*^2^ statistics to interpret the impact that the heterogeneity has in each case.

**Table VI:** Summary of heterogeneity statistics for the periodontal data in example 1

*p*	*μ*_1_	*μ*_2_	*R*	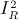	*H*^2^	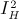	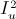	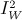
1	1	0	2.14	0.78	—	—	0.72	0.72
1	0	1	4.79	0.96	—	—	0.94	0.94
2	1	1	3.10	0.90	16.03	0.94	—	—

The variable *p* is the number of treatment effect parameters that the statistic applies to, and columns *μ*_1_ and *μ*_2_ indicate whether the statistics apply to this particular parameter. *R*, 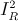, *H*^2^ and 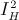 are the proposed multivariate heterogeneity statistics; 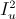 and 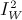 are the conventional univariate *I*^2^ statistic and White's [13] *I*^2^ statistic, respectively.

**Table VII:** Summary of heterogeneity statistics for the sleep data in example 2

*p*	*μ*_1_	*μ*_2_	*R*	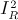	*H*^2^	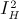	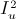	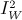
1	1	0	2.09	0.77	—	—	0.75	0.75
1	0	1	1.52	0.57	—	—	0.39	0.60
2	1	1	1.67	0.64	2.83	0.65	—	—

The variable *p* is the number of treatment effect parameters that the statistic applies to, and columns *μ*_1_ and *μ*_2_ indicate whether the statistics apply to this particular parameter. *R*, 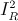, *H*^2^ and 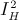 are the proposed multivariate heterogeneity statistics; 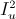 and 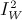 are the conventional univariate *I*^2^ statistic and White's [13]*I*^2^ statistic, respectively.

**Table VIII:** Summary of heterogeneity statistics for the MYCN and chromosome 1p data in example 3

*p*	*μ*_1_	*μ*_2_	*μ*_3_	*μ*_4_	*R*	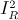	*H*^2^	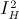	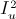	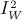
1	1	0	0	0	1.61	0.61	—	—	0.60	0.59
1	0	1	0	0	2.52	0.84	—	—	0.72	0.77
1	0	0	1	0	1.96	0.74	—	—	0.62	0.66
1	0	0	0	1	1.61	0.61	—	—	0.42	0.57
2	1	1	0	0	2.03	0.76	—	—	—	—
2	1	0	1	0	1.65	0.64	—	—	—	—
2	1	0	0	1	1.57	0.60	—	—	—	—
2	0	1	1	0	2.23	0.80	—	—	—	—
2	0	1	0	1	1.90	0.73	—	—	—	—
2	0	0	1	1	1.77	0.68	—	—	—	—
3	1	1	1	0	1.91	0.73	—	—	—	—
3	1	1	0	1	1.79	0.69	—	—	—	—
3	1	0	1	1	1.63	0.62	—	—	—	—
3	0	1	1	1	1.92	0.73	—	—	—	—
4	1	1	1	1	1.77	0.68	2.66	0.63	—	—

The variable *p* is the number of treatment effect parameters that the statistic applies to, and columns *μ*_1_ - *μ*_4_ indicate whether the statistics apply to this particular parameter. *R*, 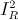, *H*^2^ and 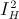 are the proposed multivariate heterogeneity statistics; 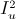 and 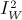 are the conventional univariate *I*^2^ statistic and White's [13] *I*^2^ statistic, respectively.

### 6.1. Example 1: the periodontal data

The standard univariate *I*^2^ and White's *I*^2^ statistics are in good agreement in Table VI. The 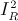 statistic for the first outcome is, however, noticeably larger than the others (0.78 compared with 0.72). This suggests that the heterogeneity may have a little more impact for the first outcome than the standard univariate statistic indicates. Interestingly, the *I*^2^ statistics relating to both outcomes jointly (*p* = 2) are almost as great as those for the second outcome alone. This suggests that the very considerable heterogeneity, coupled with the uncertainty in the estimates of the between-study covariance matrix from pooling just five studies, has more impact for the joint analysis than an average of the univariate measures might be thought to indicate. To summarise, the univariate heterogeneity statistics describe the impact of the heterogeneity for the marginal inferences quite well, as anticipated, but the multivariate heterogeneity statistics add further insight. All the *I*^2^ statistics are greater than 0.7, and some are larger than 0.9, which indicates that the heterogeneity has a considerable impact.

### 6.2. Example 2: the sleep data

The univariate and multivariate heterogeneity statistics for the first outcome in Table VII are in good agreement, but those for the second outcome differ greatly as anticipated. Because the standard univariate and White's *I*^2^ statistics for the second outcome depend on the univariate and multivariate estimates of Σ_22_, respectively, it is clear from Table III that they must be in poor agreement. Those more familiar with the standard univariate measure might suspect that White's *I*^2^ is unduly effected by the large multivariate estimate of Σ_22_, because this *I*^2^ is a function of this particular and rather extreme entry of the estimated between-study covariance matrix. Although 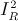 tempers White's *I*^2^ for the second outcome slightly, it confirms that the impact of the heterogeneity is more considerable for *μ*_2_ in a multivariate meta-analysis than a univariate meta-analysis, even after taking into account the entire model fit. The multivariate 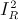 and 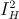 heterogeneity statistics for both treatments, and hence the joint inference and the meta-analysis as a whole, are in good agreement. The *I*^2^ statistics are not as large as those for the previous example, but they are all greater than 0.3, indicating that the heterogeneity has a quite considerable impact.

### 6.3. Example 3: the prognostic value of MYCN and chromosome 1p

White's *I*^2^ statistics are generally larger than the usual univariate *I*^2^ statistics in Table VIII, as anticipated from the larger estimated between-study variances from the multivariate model shown in able IV. However, the 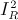 statistics for single treatment effect parameters (*p* = 1) are even larger than White's, suggesting that the impact of the heterogeneity is even greater. It seems that allowing for the uncertainty in the model fit, coupled with the relatively high dimension of the multivariate meta-analysis and the amount of missing data, means that the heterogeneity has a little more impact than either of the previously suggested *I*^2^ statistics indicate. These larger multivariate heterogeneity statistics are also apparent for the *p* > 2 statistics, which are averages of the corresponding *p* = 1 heterogeneity statistics. The general picture from Table VIII is that the impact of the heterogeneity is really quite considerable for this example, and much more so than suggested by the univariate or White's *I*^2^ statistics.

## 7. Multivariate meta-regression

The multivariate random effects model may be extended to a multivariate meta-regression model where the treatment effect vector *μ* includes study level covariate effects. For example, if the first outcome depends on a covariate *x*, we replace *μ*_1_ with *α*_1_ + *β*_1_*x*_*i*_. We refer to all parameters in the mean of (1) as treatment effect parameters, irrespective of whether they are intercept or covariate effects. The inference for the treatment effect parameters follows in an analogous way to (2) and (3); here, the model is fitted as a weighted linear regression model, where all weights are regarded as known [14].

All our heterogeneity measures are immediately applicable to a multivariate meta-regression. We can obtain the covariance matrix of the estimates of all treatment effect parameters under the assumptions of fixed and random effects meta-regression models and obtain *R* and 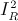 statistics for any combinations of treatment effect parameters of interest. Now that covariate effects are included, we may be especially interested in all parameters for a particular outcome, for example. Equation (B5) continues to provide the expected value of *Q*_*s*_, where the degrees of freedom *v* is the number of observations minus the total number of treatment effect parameters (intercept terms plus covariate effects). Hence, we define *H*^2^ = *Q*_*s*_ / *v* and 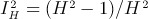 as heterogeneity statistics for a meta-regression; but, just as in meta-analysis, we only use these to quantify the impact of heterogeneity for all treatment effect parameters.

## 8. Discussion

We have proposed some multivariate measures of the impact of heterogeneity in a multivariate meta-analysis. All aspects of the data contribute to the calculations; and hence, our measures can be expected to perform well regardless of the amount of borrowing of strength involved and any vagaries of the particular dataset under consideration. A considerable advantage of our proposals is that they are relatively easily computed from standard output. A potential limitation of our proposals is that it is tempting to interpret them in an overly simplistic fashion. For example, the Cochrane handbook [6] has important things to say about the interpretation of univariate *I*^2^ statistics, and these same issues apply here. Perhaps most importantly, the Cochrane handbook makes it clear that the use of particular thresholds when interpreting heterogeneity statistics can be misleading.

Perhaps one advantage of *H*^2^ and 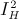 is that they both reduce to the conventional measures univariately. The multivariate *R* statistic also simplifies to the univariate *R*, but 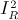 is an analogue of *I*^2^ that only simplifies to *I*^2^ univariately if all studies are the same size. 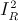 has a similar but different interpretation to the conventional univariate *I*^2^ statistic. Another advantage of *H*^2^ and 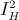 is that their computation does not require fitting the random effects model; only the fixed effects model fit is required. This eases their computation, and these multivariate heterogeneity statistics can be obtained without comparing the fixed effects results to any particular random effects model. Hence, *H*^2^ and 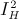 provide a means to quantify the heterogeneity concisely in situations where many possible random effects models and estimation methods are to be considered. Alternative *R* and 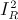 statistics are obtained for different fitted random effects models, for example, using different estimation procedures for estimating the between-study covariance matrix. Alternatively, one could fit a reduced random effects model, where perhaps all between-study variances are assumed to be identical. Irrespective of how the between-study covariance matrix has been estimated, and the assumptions made about its form, the *R* and 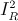 statistics may be calculated for each fitted model. This may also be considered an advantage of these statistics, however, because the impact of heterogeneity may be thought to depend on this modelling and estimation.

There is therefore no single ‘best’ multivariate heterogeneity statistic, but the meta-analyst who desires a single heterogeneity statistic, and is committed to using *I*^2^ in the univariate scenario, is likely to find the 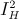 statistic appealing. However, the meta-analyst who requires a more thorough investigation into the impact of the heterogeneity, on all the various combinations of treatment effects of interest, is more likely to use *R* and 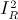.

Our measures quantify the impact of heterogeneity in an analogous way to the conventional univariate heterogeneity statistics, but the multivariate scenario allows a much richer array of possibilities. We are currently developing statistics that describe the amount borrowing of strength afforded by multivariate, rather than univariate, meta-analyses and other related quantities that might also be of interest. In addition to such possibilities, we now advocate routinely reporting the estimated between-study covariance matrix, and the covariance matrix of the estimate of the treatment effect, although we recognise that we ourselves have not always fully reported them. These quantities enable others to make use of all aspects of the model fit in any subsequent analysis and provide many insights into the properties of the fitted multivariate model. We also advocate providing our and other heterogeneity statistics as further descriptive statistics because they also add insight. For example, by comparing univariate and multivariate measures of heterogeneity, meta-analysts can directly assess the impact of heterogeneity for univariate and multivariate analyses of their data.

The uncertainty in the univariate heterogeneity measures is usually considerable, however, and this can also be expected multivariately. A related concern is that the properties of the proposed multivariate statistics are poorly understood, and we accept that these deserve further investigation and this may form the subject of a future paper. Attempting to derive multivariate confidence regions corresponding to the proposed measures is at best extremely difficult. We leave the best way to do this, and indeed the question of whether this is necessary or desirable, as an open research question, but those who require an indication of the uncertainty in their measures might consider bootstrapping. In any case, practitioners typically use the univariate heterogeneity statistics as descriptive statistics. The statistics are interpreted as measuring the impact of the heterogeneity for their particular meta-analysis, and any regard for the properties of the measures under repeated sampling is a secondary consideration.

Other multivariate measures are also possible, and a statistic similar in concept to (5), but based on the trace of the covariance matrices, may warrant consideration. Some meta-analysts use quantiles from the *t* distribution when calculating confidence intervals using the random effects model and may wish to scale up our *R* statistics by the ratio of *t* and standard normal quantiles to reflect this, which provides another variation of our methods.

The cautiously minded statistician is likely to want to perform separate univariate meta-analyses in addition to a multivariate meta-analysis, to see if the univariate and multivariate results differ qualitatively. Similarly, it may be of interest to see how the univariate heterogeneity measures compare with those proposed here. If the various heterogeneity statistics differ substantially, then this is of interest; and the reasons for this should be explored and, if possible, explained. Our proposed methods measure the impact of heterogeneity differently to the standard methods, and we think that they are preferable when multivariate meta-analysis has been used because they accurately reflect the multivariate nature of the model fit. Greater insight into the data is generally afforded by looking at the data in a variety of ways, and we hope that our methods will embellish, rather than diminish, the established ways of measuring the impact of heterogeneity in meta-analysis that we have taken as our inspiration here.

## Appendix A

Let *λ*_*R*,*i*_, *i* = 1, ⋯ *p*, denote the eigenvalues of , and let *λ*_*F*,*i*_ denote the eigenvalues of **C**_F_. The confidence ellipsoids for the random effects and fixed effects meta-effects analyses are centred at the corresponding point estimates and have axes  and , where *e*_*R*,*i*_ and *e*_*F*,*i*_ are the normalised eigenvectors corresponding to the eigenvalues *λ*_*R*,*i*_ and *λ*_*F*,*i*_ [21], p. 153; *c* is the square root of the critical point of  (usually the 0.95 percentile). The ‘volume’ of the random effects confidence ellipsoid (the length of the interval in 1-d) is therefore

(A1)

where *k*_*p*_ = 2*π*^*p* / 2^ / *p*Γ(*p* / 2); if *p* = 1, *k*_*p*_ = 2; if *p* = 2, *k*_*p*_ = *π* and if *p* = 3, *k*_*p*_ = 4*π* / 3, for example. Because the product of the eigenvalues is the determinant of a square, matrix (A1) becomes

Similarly,

The ratio of the volume of the confidence ellipsoids is *V*_R_ / *V*_F_. Combining the two equations immediately above with (4) gives (5).

## Appendix B

The univariate *H*^2^ statistic estimates the quantity 1 + *ρ* = 1 + *τ*^2^ / *σ*^2^ when all studies are the same size [5]. We can assess the properties of *Q*_*s*_, and hence what *H*^2^ estimates, by assembling all the **Y**_*i*_ into a single vector **Y** and fitting a fixed effects meta-regression model

(B1)

where **S** = diag(**S**_*i*_). **X** is the regression design matrix; if all studies provide all outcomes, **X** is a column of *p* by *p* identity matrices and *β* = *μ*. The real statistical model is

(B2)

where ***Δ*** denotes the assumed between-study covariance matrix of **Y**. This matrix is block diagonal because the studies are assumed independent, and if all studies provide all outcomes, then this matrix is diag(***Σ***).

Transform the data, **Z** = **S**^− 1 / 2^**Y**, and write **W** = **S**^− 1 / 2^**X**, so that (B1) and (B2) become

(B3)

and

(B4)

Model (B3) is an unweighted regression, and as usual, we have

where  is the vector of residuals from the regression of **Z** on **W**, **H** is the corresponding ‘hat’ matrix and *ε* are the true errors that can be obtained from (B4). We have the usual results that (**I** − **H**) is symmetrical, idempotent and its trace equals its rank, which equals the regression's degrees of freedom (*v*). The statistic *Q*_*s*_ is the sum of the squared residuals when fitting model (B3), but model (B4) is assumed correct so

Using the properties of (**I** − **H**) and interchanging trace and expectation operations, we evaluate the expectation of *Q*_*s*_ as

(B5)

so that

Hence, the multivariate *H*^2^ statistic, like its univariate counterpart, is a measure of the relative excess of *Q*_*s*_ over its degrees of freedom, where this excess is measured by the trace of a matrix that takes into account the model fit, via **I** − **H**, and the relative magnitude of the within-study and between-study variance matrices.

If all studies provide all outcomes and are the same ‘size’, then we have **S** = diag(**S**_1_), ***Δ*** = diag(***Σ***), and

where **J** is an *np* by *np* matrix where all submatrices are *p* by *p* identity matrices. Also, the degrees of freedom become *v* = *np* − *p* = (*n* − 1)*p*. Hence,

so that

and  is a measure of , which is an appropriate generalisation of the univariate measure *ρ* / (1 + *ρ*). The trace of a covariance matrix is another useful scalar summary statistic for the dispersion of a multivariate random variable (it is the sum of the variances and sometimes referred to as the total variance), so this is appropriate. For example, fixed effects model corresponds to ; if the between-study and within-study covariance matrices are the same, then this corresponds to , and so on. The formulae for *E*(*Q*_*s*_) show that *H*^2^ and  measure a suitable quantities, but, unless all studies are the same size, these quantities have less intuitive appeal than those that *R* and  measure.

## Appendix C

We assume that **S**_*i*_ is positive definite for all *i* and that  has been constrained to be positive semi-definite, so that  is positive definite. All these matrices are Hermitian matrices (because they are symmetric and their entries are real numbers). We write  to indicate that  is positive semi-definite, which is equivalent to . We write **A** *≥***B** to mean that **A** − **B** *≥*0 so that

and hence, using Corollary 7.7.4(a) from Reference [22], p. 471,

so that, because the matrices on both sides of the aforementioned equation are also Hermitian, and using the property described in Problem 2 from Reference [22], p. 475,

so that

which, provided that (3) is used to evaluate , is the same statement as

(C1)

Using the positive semi-definite analogue of Observation 7.1.2 from Reference [22], p. 397, which means that any principal submatrix of a positive semi-definite matrix is also positive semi-definite, we can take  and **C**_F_ in (C1) as referring to all or a subset of the outcomes as desired. Finally, using Corollary 7.4.4(b) of Reference [22], p. 471, we have that

so that *R ≥*1 in (5) and  in (6).
